# Evolution of superficial spreading melanoma to resemble desmoplastic melanoma: case report

**DOI:** 10.1007/s00428-021-03127-0

**Published:** 2021-07-20

**Authors:** Martin G. Cook, Barry W. E. M. Powell, Megan E. Grant, Adele C. Green

**Affiliations:** 1 Department of Histopathology, Royal Surrey County Hospital, Egerton Road, Guildford, GU2 7XX UK; 2 Molecular Oncology Group, Cancer Research UK Manchester Institute, University of Manchester, Wilmslow Road, Manchester, M20 4BX UK; 3 Division of Clinical Medicine, University of Surrey, Guildford, GU2 7XH Surrey UK; 4 Department of Plastic and Reconstructive Surgery, St. George’s Hospital, Blackshaw Road, Tooting, London, SW17 0QT UK; 5 QIMR Berghofer Medical Research Institute, Brisbane, QLD 4006 Australia

**Keywords:** Desmoplastic melanoma, Superficial spreading melanoma, Mixed desmoplastic, Tumor behavior reversal

## Abstract

Desmoplastic melanoma commonly occurs on the head and neck in a pure form, but occasionally, it occurs in a mixed tumor with another type, usually superficial spreading melanoma (SSM), and rarely as a metastasis from a primary SSM. We report here a primary SSM on the leg of a 32-year-old male which metastasised to lymph nodes, and 10 years later recurred at the primary site initially with mixed features but evolving to resemble a uniformly desmoplastic, deeply invasive melanoma. This unusual case has implications for clinical management and is additionally notable for its reversal in behavior, from metastatic to local infiltrative type, correlating with the change in morphology.

Desmoplastic melanoma most commonly occurs on the head and neck as a poorly circumscribed dermal mass and tends to recur locally rather than metastasise [1]. It usually occurs in pure form but occasionally is seen as a desmoplastic component of a mixed tumor with the other element being a lentigo malignant melanoma (LMM) or superficial spreading melanoma (SSM) type and behavior then relates to the more aggressive component [2]. Very rarely a metastasis from SSM occurs as a desmoplastic lesion [3, 4]. We describe here a primary melanoma of clear SSM phenotype which metastasised to lymph nodes and showed in-transit metastases, but 10 years later recurred near the scar at the primary site with mixed conventional and desmoplastic features and then progressed to resemble a completely desmoplastic, deeply invasive melanoma at the original site. We believe it important to record this unusual case as it has implications for clinical management. In addition, we note that the reversal in aggressive behavior from metastatic to local infiltrative type was correlated with the change in morphology.

## Case report

In 2005, a male aged 32 years was referred by his general practitioner to a plastic surgeon with a newly acquired pigmented lesion on the left calf suspected to be melanoma. This was excised and histologically diagnosed as SSM, 1.5-mm thick, with mitotic count 6/mm^2^. It was widely excised and sentinel node biopsy was performed. Two sentinel nodes from the left groin both contained a parenchymal metastasis 0.2 mm in maximum dimension. The melanoma was shown to have a BRAF^V600E^ mutation.

**Fig. 1:**
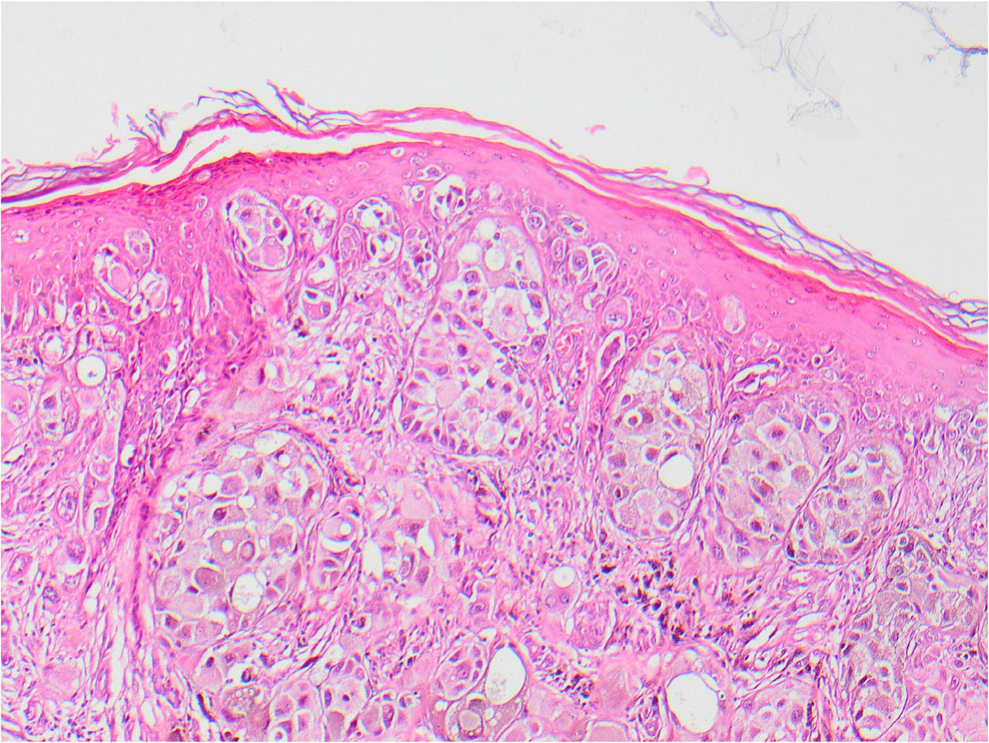
The superficial part of the presenting melanoma in 2005 on the left leg showing pagetoid infiltration, characteristic of SSM

**Fig. 2:**
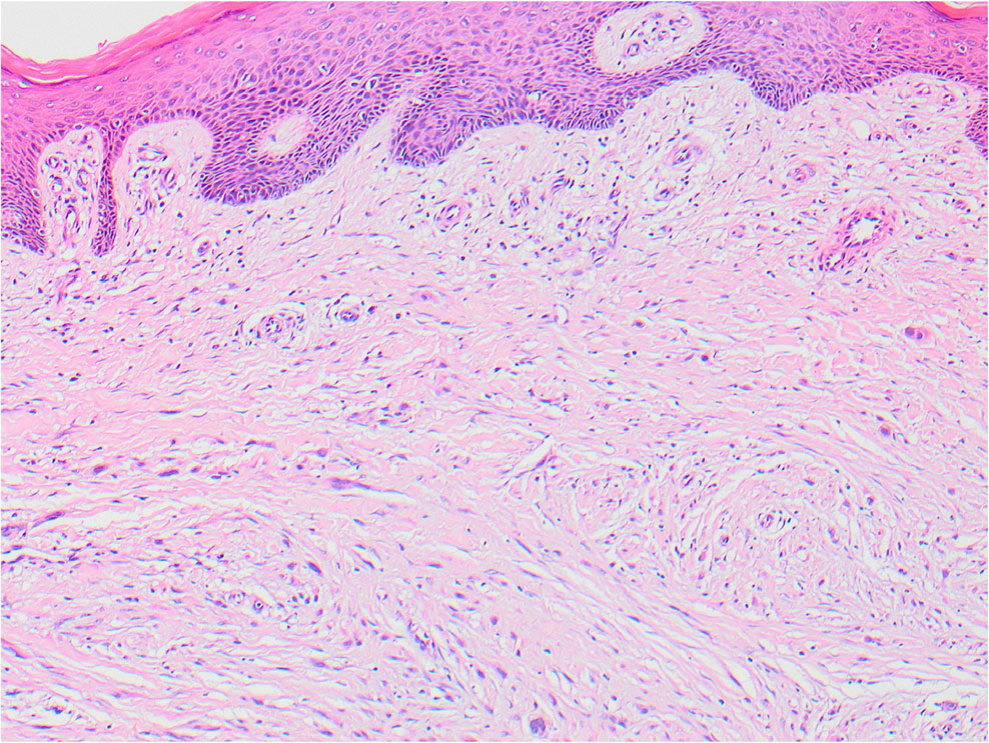
The skin over the excised lesion in 2015 on the left leg, 10 years later showing the dermal proliferation of spindle cells with desmoplasia

**Fig. 3:**
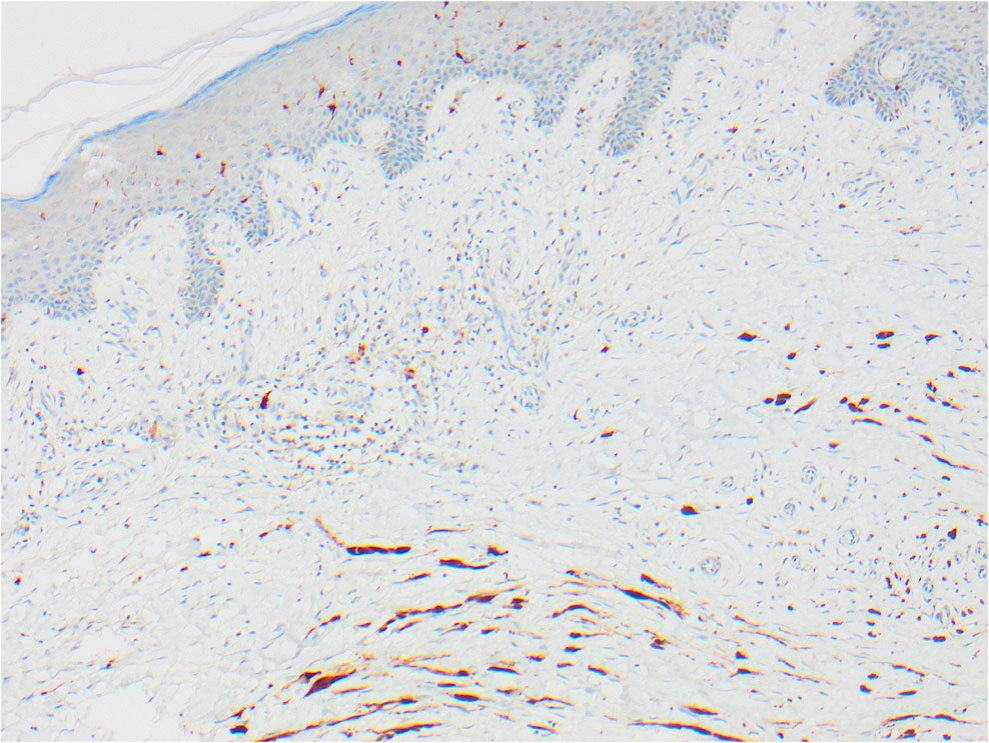
The skin seen in Fig. 2 shows elongated spindle cells positive with S100

In the same year, 2005, the patient had six clinically benign moles on the mid- and upper back excised, one of which was histologically melanoma in situ. In 2008, three further pigmented lesions were removed from the left anterior calf, left lateral thigh, and left anterior shin. All were confirmed as benign junctional naevi, completely excised. However, in 2009, 4 years after diagnosis of the primary SSM, the patient found a nodule in the upper left popliteal fossa. Histology showed completely excised metastatic melanoma. In 2011, 6 years after diagnosis of the primary SSM, new pigmentation was noted adjacent to the original scar, and on excision was histologically diagnosed as a junctional naevus with dysplasia without a dermal component (Figs. 1, 2, 3).

In 2015, 10 years after excision of the primary SSM on the calf, three subcutaneous nodules were identified near the original scar. On excision, these were shown to be recurrent melanoma with an epithelioid component similar to the original, but merging with a spindle cell desmoplastic component. The deeper spindle desmoplastic component was S100-positive but HMB45-negative, in contrast to the original melanoma which was HMB45-positive.

In August 2016, a further subcutaneous lesion was identified on the left calf underlying the previous scar. This was excised and described histologically as spindle cell recurrent melanoma. Ten months later in June 2017, and 12 years after diagnosis of the original primary SSM, another excision of a further subcutaneous lesion near the original scar showed merging of atypical spindle cells with scarring suggesting progression to desmoplastic melanoma. In April 2018, another nodule excised near the scar showed features of desmoplastic melanoma extending to the margins of the specimen. A further excision in May 2018 contained more desmoplastic melanoma still showing a BRAF^V600E^ mutation, and this time appearing completely excised. In September 2020, the patient was referred to another hospital with intrathoracic lesions radiologically suspected as metastases. A biopsy was not proposed as the features were so highly consistent with metastatic melanoma.

## Discussion

The histological interest of this case rests on the evolution over a 10-year period from primary SSM with nodal metastases to a spindle cell tumor with desmoplasia and without further metastasis but recurring locally with features of desmoplastic melanoma. The earlier metastases of epithelioid-type melanoma were to a groin node and an in-transit metastasis in the popliteal fossa. These were completely excised.

Ten years after the original excision, the tumor recurred in the original scar with mixed epithelioid and spindle cell features and this then recurred again with spindle/desmoplastic features 13 years after the original presentation. It had the infiltrative pattern of a desmoplastic melanoma and a further complete excision was achieved. No perineural invasion or neutropism was identified. Not only did the histological features change with time but also the pattern of growth also changed from metastatic to locally infiltrative, although it still retained a BRAF mutation which is unusual in desmoplastic melanoma. Their molecular features typically include NF1 mutations as well as in CBL, ERBB2, MAP2K1, and MAP3K1 [5–7]. It appears that this melanoma underwent metaplastic change to resemble desmoplastic melanoma and acquired the associated biological characteristics, but it retained the BRAF mutation and eventually has reverted to biological behavior more typical of SSM.

The rate of mutations is higher than in most other melanomas, and the majority are said to be related to ultraviolet radiation mutagenesis [5]. Whether this applies to the large minority of desmoplastic melanomas that arise on infrequently exposed anatomical sites remains to be established, as does the genetic signature in mixed desmoplastic and other melanoma types.

## Data Availability

Not applicable.

## References

[1] de Almeida LS (2008). Desmoplastic malignant melanoma: a clinicopathologic analysis of 113 cases. Am J Dermatopathol.

[2] George E (2009). Subclassification of desmoplastic melanoma: pure and mixed variants have significantly different capacities for lymph node metastasis. J Cutan Pathol.

[3] Busam KJ (2004). Cutaneous desmoplastic melanoma: reappraisal of morphologic heterogeneity and prognostic factors. Am J Surg Pathol.

[4] Scolyer RA, Thompson JF (2005). Desmoplastic melanoma: a heterogeneous entity in which subclassification as “pure” or “mixed” may have important prognostic significance. Ann Surg Oncol.

[5] Rabbie R (2019). Melanoma subtypes: genomic profiles, prognostic molecular markers and therapeutic possibilities. J Pathol.

[6] Kim J (2012). BRAF, NRAS and KIT sequencing analysis of spindle cell melanoma. J Cutan Pathol.

[7] Davison JM (2005). Absence of V599E BRAF mutations in desmoplastic melanomas. Cancer.

